# The Importance of Confirming False Homozygosity in Pretransplant HLA Typing Results of Patients with Hematologic Malignancies

**DOI:** 10.7150/ijms.99883

**Published:** 2024-09-16

**Authors:** Min Young Lee, Eunkyoung You

**Affiliations:** 1 Department of Laboratory Medicine, Kyung Hee University College of Medicine and Kyung Hee University Hospital at Gangdong, Seoul, Korea.; 2 Department of Laboratory Medicine, Inje University College of Medicine, Busan Paik Hospital, Busan, Korea.

**Keywords:** Human leukocytic antigen (HLA), Loss of heterozygosity (LOH), Acute myeloid leukemia (AML), Hematopoietic stem cell transplantation, Homozygosity, Chromosomal microarray

## Abstract

Loss of heterozygosity (LOH) on chromosome 6p, where the HLA genes are located, can result in incorrect homozygosity findings during HLA genotyping in patients with hematologic malignancies. The degree of HLA compatibility between donor and recipient is crucial in hematopoietic stem cell transplantation. Therefore, we present a case of false homozygosity in HLA genotyping due to LOH on chromosome 6p in a patient diagnosed with acute myeloid leukemia (AML).

HLA molecular typing was conducted on both peripheral blood and buccal swab samples. The analysis included sequence-based typing (SBT) and next-generation sequencing-based typing. Additionally, chromosomal microarray analysis (CMA) was performed.

A 68-year-old male presented with anemia and thrombocytopenia. Subsequent bone marrow examination confirmed AML. High-resolution HLA genotyping of Peripheral blood during blast crisis revealed homozygosity at the -A, -B, and -C loci. Conventional karyotyping showed a normal karyotype, 46,XY[20]. Retesting of HLA genotyping one week later confirmed the homozygous results. Subsequently, HLA typing was repeated using buccal swab specimens, confirming heterozygosity at all 4 HLA loci. CMA on peripheral blood samples during blast crisis revealed a large terminal region of copy-neutral LOH spanning approximately 43.5 Mb in the chromosome region 6p25.3p21.1.

LOH at the HLA gene locus can significantly impact donor selection, potentially leading to the selection of mistakenly identified homozygous donors. Clinicians and laboratory personnel should be aware of these issues to prevent erroneous HLA typing results in patients with hematologic malignancies. It is advisable to confirm the HLA typing of recipients with hematologic malignancies whenever homozygosity is detected at any locus. This can be achieved through careful interpretation of low peaks in SBT, and by using buccal swab samples or peripheral blood collected after achieving remission.

## Introduction

Hematopoietic stem cell transplantation (HSCT) is a pivotal therapeutic option in the treatment of acute myeloid leukemia (AML), a hematologic malignancy characterized by the rapid proliferation of abnormal myeloid cells inhibiting the production of normal hematopoietic cells. The success of HSCT is determined by the compatibility of human leukocyte antigens (HLA) between the donor and the recipient. An identical HLA match was deemed the conventionally ideal method, usually sourced from a sibling or close relative or unrelated registry donor. Therefore, in the treatment of AML, HLA typing plays a crucial role in increasing the success rate of transplantation, reducing complications and selecting the optimal donor.

Copy neutral-loss of heterozygosity (CN-LOH) is an alteration in malignancies that helps evade immune detection, also prevalent in hematologic malignancies. When CN-LOH occurs on chromosome 6p, which harbors the major histocompatibility complex (MHC) region, it can lead to false homozygosity in HLA typing. This can give rise to a serious problem in hematopoietic stem cell transplantation in that the patient would be at a risk of being transplanted from a falsely matched donor if the false typing results are not properly recognized. While such occurrences are often observed after HSCT or at relapse of leukemia, it is rare to detect them at initial diagnosis.

We report a case where homozygosity was discovered in HLA typing performed on peripheral blood (PB) at the diagnosis of AML, and subsequent laboratory tests confirmed CN-LOH. Furthermore, we aim to review cases where CN-LOH was identified at the diagnosis of hematologic malignancies, emphasizing the necessity to consider CN-LOH as a potential complicating factor during the diagnostic process. This review will include methodologies for detecting CN-LOH.

## Materials and methods

### Sources of DNA

The patient's molecular typing was conducted using DNA extracted from PB and buccal swabs (BS) with a Qiagen kit (QIAGEN, Hilden, Germany). The concentration and purity of DNA were determined using a spectrometer by measuring the absorbance ratio at 260 nm and 280 nm wavelengths (NanoDrop One Spectrophotometer, ThermoFisher Scientific, Wilmington, DE, USA). In all samples, the A260/A280 ratio fell within the range of 1.8-2.0.

### HLA typing

High-resolution typing at the specified loci was carried out using a commercial sequence based typing (SBT) kit, AVITA plus HLA SBT kits (Biowithus, Seoul, Republic of Korea). Nucleotide sequences were acquired using the ABI 3500 Dx Genetic Analyzer (Thermo Fisher Scientific, Waltham, Massachusetts). Subsequently, the generated sequences were analyzed using the BIOWITHUS SBT Analyzer (Biowithus).

### Next generation sequencing (NGS)

NGS was conducted to validate the high-resolution typing results. In brief, HLA-A, -B, -C, -DRB1, and -DQB1 loci were amplified from purified sample DNA using the AllType reagent kit (One Lambda, Inc., Canoga Park, CA, USA). Following amplification, sample libraries were prepared using Ion 520^TM^ and 530^TM^ExT kits (One Lambda, Inc., Canoga Park, CA, USA). The final prepared libraries underwent sequencing using an IonTorrent S5 sequencer (One Lambda, Inc., Canoga Park, CA, USA) on an IONS5-520 chip (One Lambda, Inc.). Analysis of the data was performed using One Lambda's TypeStream Visual 3.0 NGS Analysis software.

### Chromosomal microarray (CMA)

CMA was performed on genomic DNA extracted from PB using a whole genome platform that included both copy number (CN) and SNP probes on an Affymetrix CytoScan Dx Assays (Affymetrix, Santa Clara, CA, USA) according to manufacturer's protocols. This platform includes 550,000 CNV markers and 200,000 SNP markers with an average resolution of 100 kb. The data were visualized and analyzed using the Chromosome Analysis Suite software package (Affymetrix). The February 2009 human reference sequence (GRCh37/Hg19, http://genome.ucsc.edu/) was used as the reference sequence.

### Samples collected from the patient and tests performed

Sample #1 of PB was collected in an EDTA tube on August 26, 2022, and DNA extraction was performed on the same day. This sample was tested with the CMA assay. Sample #2 of PB was collected in an EDTA tube on September 02, 2022, and DNA extraction was carried out on the same day. The BS sample (sample #3) was received on September 08, 2022, and DNA was isolated on the same day.

### Literature review

To further characterize cases of false homozygosity in HLA results associated with hematologic malignancies, a comprehensive literature review was conducted using the PubMed database. The search strategy employed the following key terms: "False homozygosity", "HLA", “LOH”, “leukemia” and “pretransplant”. This search was designed to identify relevant case reports and studies documenting instances of false homozygosity in patients diagnosed with hematologic malignancies.

## Results

### Case description

A 68-year-old Asian male with AML was HLA typed as a candidate for allogeneic HSCT. A bone marrow (BM) biopsy performed on August 25, 2022 was consistent with the diagnosis of AML, with blasts accounting for 27% of total cellularity, and cytogenetic analysis revealed a normal karyotype (46, XY). A complete blood cell count on the same day also showed that the platelet count was 58.0 × 10^9^/L, the hemoglobin was 75 g/L, and the white blood cell count was 5.53 × 10^9^/L, of which 26% were blasts. Fusion gene was negative, and next generation sequencing showed a mutation of *NPM1, KRAS, NRAS, PTPN11*, and *KMT2A* gene. The patient received transfusion irradiated pre-storage filtered packed RBCs 3 unit due to anemia. A PB sample (sample #1) was drawn on next day (August 26, 2022) for HLA typing. A complete blood cell count on the same day also showed that the platelet count was 61.0 × 10^9^/L, the hemoglobin was 98 g/L, and the white blood cell count was 5.87 × 10^9^/L, of which 24% were blasts.

The patient received chemotherapy on August 30, 2022, which consisted of 7 days of cytarabine continuously administered at 100 mg/m^2^ and idarubicin given in 3 daily doses at 12 mg/m^2^. Sample#2 was collected on September 02, 2022 while a complete blood cell count on the same day showed that the platelet count was 47.0 × 10^9^/L, the hemoglobin was 95 g/L, and the white blood cell count was 2.22 × 10^9^/L with no blasts. A buccal sample (sample #3) was collected on 6 days after sample #2 while a complete blood cell count on the same day showed that the platelet count was 24.0 × 10^9^/L, the hemoglobin was 83 g/L, and the white blood cell count was 1.41 × 10^9^/L with no blasts. Thirty days after diagnosis, a second BM biopsy demonstrated hypocelluar marrow with clearance of blasts.

### HLA typing

**Figure 1:**
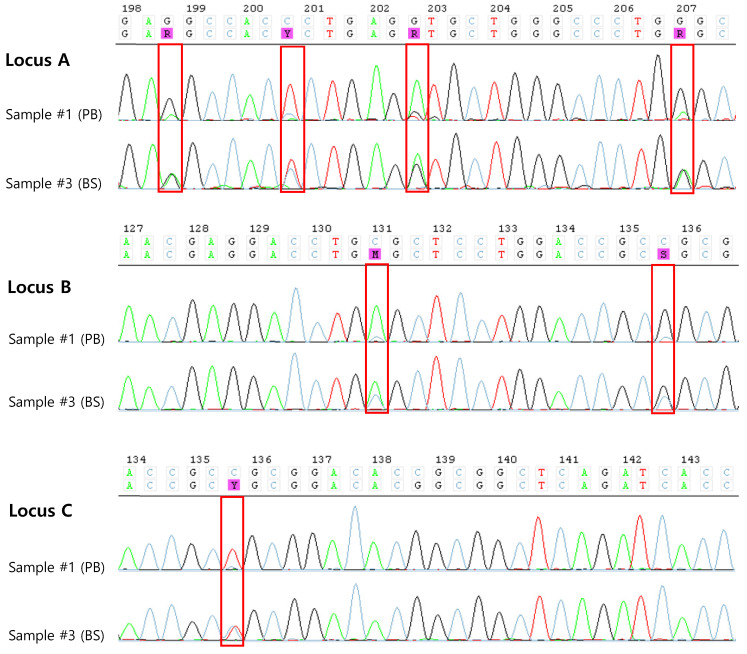
** SBT of HLA-A,-B, and -C genes from PB (sample #1) and BS (sample #3).** Chromatogram results from SBT showing very low peaks in sample #1, indicating potential heterozygosity or background noise, while sample #3 exhibits clear heterozygosity (highlighted in red box).

The results of HLA typing of the samples taken from the patient are shown in Table 1. SBT typing on sample #1 demonstrated homozygosity at the -A, -B, and -C loci except -DRB1 loci. However, a very low peak was observed in the sequence chromatogram, so the possibility of heterozygosity could not be ruled out (Figure 1). Accordingly, 7 days later, PB which was collected during chemotherapy (sample #2) and retested, and the same homozygosity results as sample #1 were confirmed. Finally, a BS sample (sample #3) obtained from the patient 6 days later collection sample #2, however, demonstrated heterozygosity at all loci tested with SBT.

**Figure 2:**
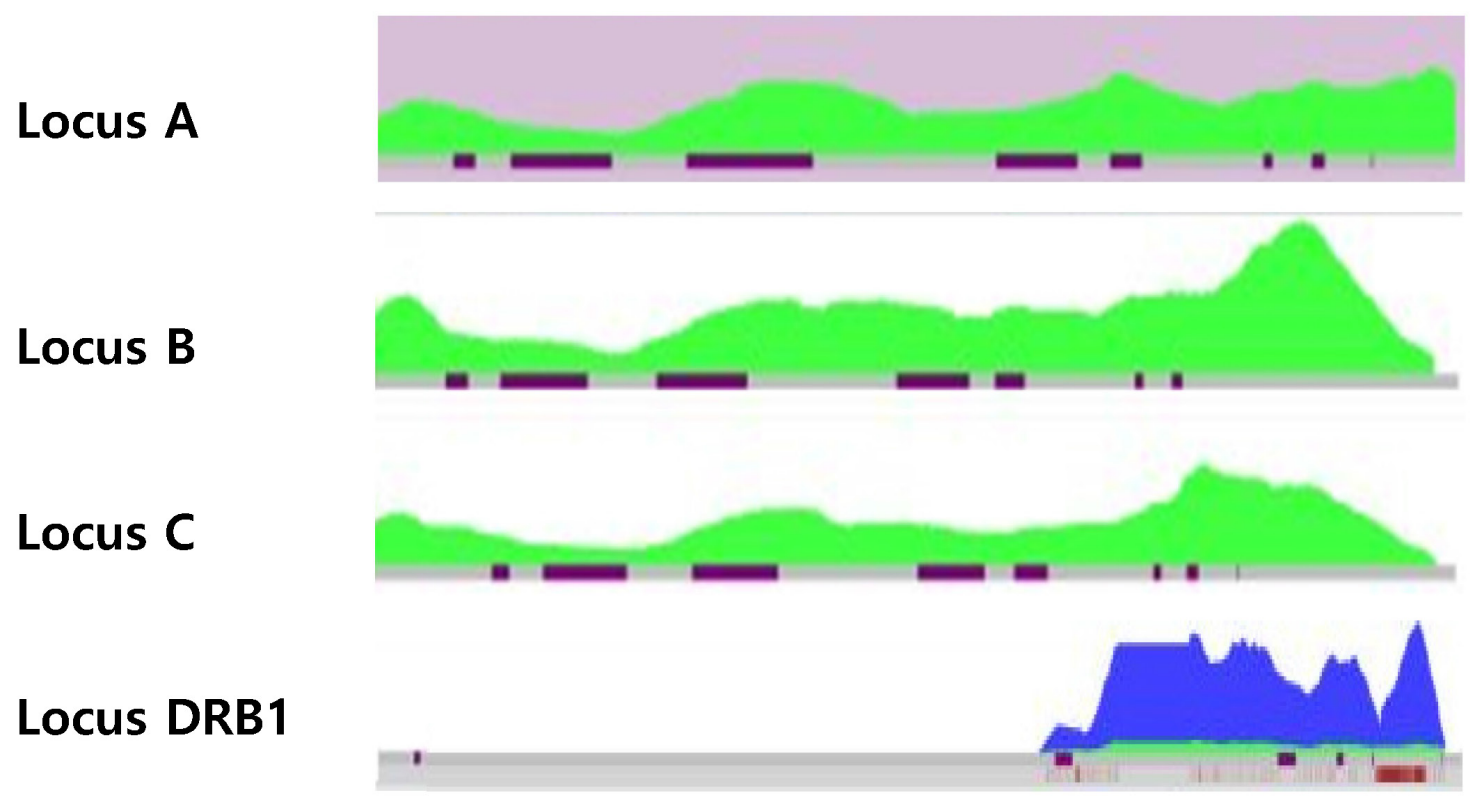
** Heat maps of sample #1 by NGS.** The histograms show depth of coverage vs. observed allele ratios in NGS data. The green plot area is allele 1 and the blue area is allele 2. Heat map shows only green color (one allele) for the sample #1 demonstrating LOH at HLA-A, -B, and -C loci. Heat map of HLA-DRB1 loci showed two colors, but allele 1 exhibited very low coverage.

We retrospectively retested sample #1 using NGS to check for differences depending on the testing method. NGS histogram results showed that, only one allele coverage was shown in sample #1 (Figure. 2).

### CMA

**Figure 3:**
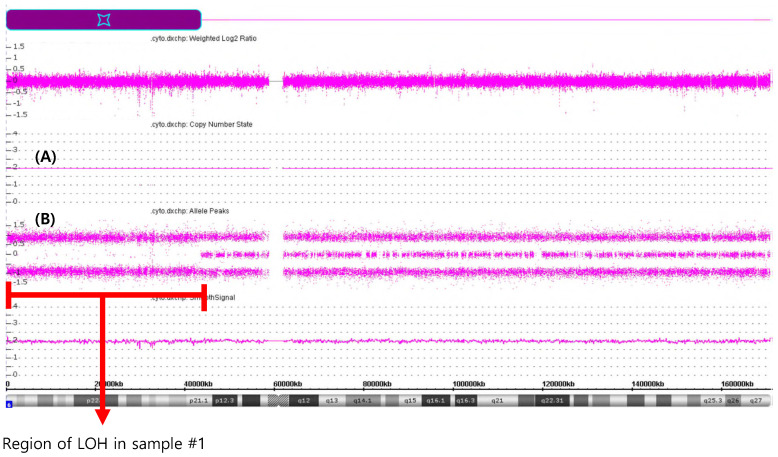
** CMA analysis of DNA extracted from PB of samples #1.** (A) solid lines represent CN state and indicate no CN changes; (B) panel represent SNP data indicating deletion of approximately 43.5 Mb located in short arm of chromosome 6.

The CMA performed on sample #1 revealed a large terminal region of copy-neutral LOH involving chromosome region 6p25.3p21.1(184,718-43,635,395), spanning approximately 43.5 megabases in size (Figure 3). This region of LOH encompassed the entire HLA gene family locus. There are also other concomitant chromosome abnormalities including gain of 11p15.5p14.2 (230,615-26,929,451)47294243), spanning approximately 26.7 megabases.

### Literature review of loss of HLA heterozygosity in hematologic malignancies at the diagnosis

In cases where HLA LOH was observed at diagnosis, the median percentage of leukemic blasts in PB was 86%. All tests were conducted using PB samples. BM karyotype analysis showed normal results in all cases except one. HLA homozygosity was identified using various methods including Single Specific Oligonucleotide (SSO), Sequence-Specific Priming (SSP), SBT, and NGS. LOH was confirmed through repeated testing after treatment, retyping using different specimens such as buccal swabs, different testing methods, or CMA. Cases with *NPM1* or *FLT3* mutations accounted for more than half of the total, comprising 64% of all cases.

## Discussion

We report a case of HLA CN-LOH detected at diagnosis in a patient with AML. HLA LOH is common in solid tumors, detected in various tumor types of 10-55% 7-9. HLA LOH is also found in hematologic malignancies, though less frequently. According to a report from a laboratory, LOH in the HLA region was found in 0.4% of HSCT patients 3. Another study on cytogenetically normal AML patients, acquired uniparental disomy on 6p21 was observed in 1.27% patients 10. However, the frequencies mentioned in the aforementioned reports pertain to all instances, irrespective of occurrence at diagnosis or relapse in hematologic malignancies. Although there are no precise reports, LOH cases are relatively more frequent after haplo-HSCT or at relapse, and rarely found at diagnosis. Consequently, the likelihood of obtaining erroneous results from CN-LOH affecting HLA at diagnosis is even rarer.

In cases of hematologic malignancies diagnosed with normal karyotype or without aberrations involving chromosome 6p, there is a lack of awareness regarding the necessity to suspect false homozygosity when HLA homozygosity results are obtained. Early detection of HLA LOH at the diagnosis can significantly impact treatment decisions, particularly those related to donor selection for HSCT. Additionally, recognizing LOH at this stage may provide insights into the pathophysiology of AML onset and progression, potentially affecting prognostic outcomes. In relapsed cases of leukemia after HSCT, the occurrence of HLA LOH and its associated prognosis and therapeutic strategies have been elucidated. Following HSCT, HLA LOH in relapsed leukemia is a critical mechanism for immune evasion, allowing leukemia cells to escape attacks from allogenic T cells and continue survival and proliferation. This process can weaken graft-versus leukemia effects, thereby increasing relapse and worsening prognosis. In such scenario, novel immunotherapies like CAR-NK cell treatment can be viable treatment options, and immune checkpoint inhibition blockade is reported to be effective 11, 12. Further research is needed to determine whether these treatments can be effectively used in cases with HLA LOH at the time of diagnosis.

As noted in previously reported cases, if homozygosity is found in HLA typing performed on PB samples containing blast cells, it is necessary to suspect LOH. In our case, very low peaks in the sequence chromatogram were observed during HLA typing, suggesting the possibility of heterozygosity. In particular, when analyzing the very low peaks observed in our case, it was found that these low peaks appeared across all exons. When these low peak positions were analyzed, the results aligned exactly with heterozygous results for HLA-A*24:02,*02:01 (low peak), HLA-B*52:01,*48:01 (low peak), and HLA-C*12:02,*08:01 (low peak). Unlike the non-specific background noise distribution, the presence of second alleles in the low peak regions suggested the need for further evaluation. We considered that these results might be due to sample contamination, interference from previous transfusions, or false homozygosity due to LOH. We tested sample #2 and confirmed the same results, concluding that the possibility of contamination or interference from transfusions was low. Therefore, we could more strongly suspect the possibility of false homozygosity due to LOH. We confirmed false homozygosity by performing a confirmatory test using a BS sample. Thus, when conducting HLA typing for hematologic malignancy patients for HSCT, caution is needed when interpreting homozygosity results. First, it is important to check whether a low peak is observed in SBT testing and whether this peak matches specific alleles when analyzed. If the peak is non-specific, the decision to report the results can be made according to laboratory guidelines. However, if the peak suggests heterozygosity, it is strongly recommended to consider the possibility of false homozygosity. In such cases, it is essential to confirm the test results using multiple methods. These methods include using alternative sample types, or retesting with a sample obtained after induction chemotherapy, where blast clearance has been achieved. However, contrary to the assertions in the reported cases, our case still showed homozygosity in HLA-A, B and C loci even when SBT was performed on PB samples without blasts after chemotherapy, similar to the HLA results obtained at diagnosis. While it is known that HLA LOH tends to occur when blast cells are present in high proportions in the sample, our case had only 26% blasts at initial diagnosis. Moreover, LOH was confirmed even in samples without blasts. There is no clear data on the number of blasts or malignant cells that can affect HLA typing in cases of LOH. Regarding the false homozygosity result in our case despite the low blast proportion, we considered the possibility that the patient might have had a predisposing disease such as myelodysplastic neoplasm (MDS) before developing AML. Dysplasia was observed in the erythroid and myeloid lineages in the patient's BM aspiration. However, the patient did not meet the diagnostic criteria for AML, myelodysplasia related. Additionally, because the patient had never visited our hospital before, there was a limitation that there was no previous examination record.

Studies have suggested that the cutoff limit for detecting heterozygous DNA in HLA typing varies by method. Dubois et al. reported that the detection level of heterozygous combinations in the Luminex microbeads assay is 15%, implying that HLA misassignment may occur when the blast percentage exceeds 80% 2. For SBT, it has been reported that the cutoff limit for the detection of heterozygous DNA is 20% through DNA mixing experiments 13. The sensitivity of different HLA typing methods varies, with SSP detecting 2.5%, reverse SSO 5-17%, and SBT 17-33% of minor alleles for heterozygosity detection 3. While SSP may only cause HLA misassignment due to LOH at over 95% blasts, SBT can cause HLA mistyping at lower blast fractions. Furthermore, there is an assertion that the cutoff of detection obtained from mixing DNA experiments should not be directly equated to the number of blasts. This is because blasts generally have a larger nucleus-to-cytoplasm ratio compared to normal leukocytes, meaning the DNA extracted from blasts could constitute a significantly larger portion than from normal leukocytes 5. The detection of LOH even after blast clearance in AML patients has not been previously reported, and the cause remains unknown. *FLT3* and *NPM1* mutations often originate in hematopoietic progenitor cells, which can differentiate into various types of blood cells. And in cases of acute leukemia that have progressed from MDS, clonal expansion of mutated hematopoietic cells in leukocytes other than blasts could be considered a potential cause.

In conclusion, HLA LOH, which is known to arise as an immune evasion mechanism during relapse, can also be detected at the time of diagnosis in de novo AML. Therefore, HLA typing for HSCT in AML patients should ideally be performed on samples collected after the clearance of blasts following treatment. However, if HLA homozygosity is observed even after blast clearance, the possibility of CN-LOH should be considered, warranting retesting or additional confirmatory tests. Specifically, when low peaks are observed in HLA typing SBT tests for hematologic malignancy patients, careful interpretation is needed to determine if the presence of other alleles is indicated, which can aid in distinguishing false homozygosity.

## Figures and Tables

**Table 1:** Result of HLA typing.

	Sample #1 (PB)		Sample #2 (PB)	Sample #3 (BS)
DATE	26/08/2022		02/09/2022	08/09/2022
CBC (blast%)	5870-9.8-61K (26%)		2220-9.5-47K (0%)	1410-8.3-24K (0%)
HLA typing loci	SBT	NGS	SBT	SBT
A	*24:02	*24:02	*24:02	*24:02
	*24:02	*24:02	*24:02	*02:01
B	*52:01	*52:01	*52:01	*52:01
	*52:01	*52:01	*52:01	*48:01
C	*12:02	*12:02	*12:02	*12:02
	*12:02	*12:02	*12:02	*08:01
DRB1	*09:01	*09:01	*09:01	*09:01
	*15:02	*15:02	*15:02	*15:02

PB, peripheral blood; BS, buccal swab; SBT, sequence based typing; NGS, next-generation sequencing.

**Table 2:** Published cases of loss of heterozygosity HLA results in hematologic malignancies at the diagnosis

Case #	Diagnosis	Blast % (PB/BM)	Specimen	Chromosome BM	Mutations	HLA typing	HLA loci with LOH	Methods for confirmation of LOH	LOH region
1^1^	AML	91%/ N/A	PB	Normal	N/A	SSO, SSP	-A, -B	SBT in remission	N/A
2^2^	AML M1	95%/ 96%	PB	Normal	*FLT3, WT1, NPM1*	SBT, SSO	-A, -B, -C, -DRB1, -DQB1	SSO, SSP and SBT after treatment	N/A
3^2^	AML M5b	96%/ N/A	PB	Normal	*FLT3, WT1*	SBT, SSO	-A, -B, -C, -DRB1	SSO, SSP and SBT after treatment	N/A
4^2^	AML M5a	85%/ N/A	PB	Normal	*FLT3 weak, NPM1*	SBT, SSO	A, -B, -C,	SSO, SSP and SBT after treatment	N/A
5^2^	AML M1	N/A/ N/A	PB	Normal	*FLT3*	SBT, SSO	-A, -B, -C	SSO, SSP and SBT after treatment	N/A
6^2^	AML M1	96%/ N/A	PB	Normal	*FLT3*	SBT, SSO	-A, -B, -C, -DRB1, -DQB1	SSO, SSP and SBT after treatment	N/A
7^2^	AML M4	27%/ N/A	PB	Y loss	*FLT3, NPM1*	SBT, SSO	-A	SSO, SSP and SBT after treatment	N/A
8^3^	AML	90%/ N/A	PB	Normal	*NPM1*	SSO, SSP	-A, -B, -C	HLA typing with BS; SSP and SBT after remission	N/A
9^3^	FL transformed into leukemic DLBCL	65%/ N/A	PB	t(14;18),+ i(14q),+18	N/A	SSO, SBT, SSP	-B, -C	SSP	N/A
10^3^	AML	79%/ N/A	PB	Normal	N/A	SSO, SBT, SSP	-A, -C	SSP	N/A
11^4^	AML	89%/ 89%	PB	Normal	N/A	SSO, SBT, NGS	-A, -B, -C, -DRB1, -DQB1	HLA typing with BS; SSO, SBT and NGS after CTx, CMA	6p25.3p21.31 (184,718-36084.672) (36Mb)
12^5^	T-ALL	87%/ 85%	PB	Normal	*SIL-TAL1, SH2B3, TCRβ,* and *TCRγ* rearrangement	SBT, NGS	-B, -C, -DRB1, -DPB1	NGS; SBT and NGS after CTx; CMA	6p25.3p21.31 (203877_33528424) (33.32Mb)
13^6^	Therapy-related AML	98%/ 93%	PB	Normal	*IDH1, NPM1*	NGS	-A	SSO and NGS after CTx; CMA	6p25.3p21.33(184,719_30,798,988) (30.6Mb)
14 *	AML	26%/ 27%	PB	Normal	*NPM1, KRAS, NRAS, PTPN11, KMT2A*	SBT, NGS	-A, -B, -C	NGS; HLA typing with BS; CMA	6p25.3p21.1(184,718-43,635,395) (43.5Mb)

PB, peripheral blood; BM, bone marrow; LOH, loss of heterozygosity, AML, acute myeloid leukemia; N/A, not available; SSO, sequence-specific oligonucleotide; SSP, sequence-specific primer; SBT, sequence based typing; BS, buccal swab; FL, Follicular lymphoma; DLBCL, diffuse large B cell lymphoma; CTx, chemotherapy; NGS, next-generation sequencing; CMA, chromosomal microarray. *present study
